# Optimizing Texture Modified Foods for Oro-pharyngeal Dysphagia: A Difficult but Possible Target?

**DOI:** 10.3389/fnut.2018.00068

**Published:** 2018-08-07

**Authors:** Samir G. Sukkar, Norbert Maggi, Beatrice Travalca Cupillo, Carmelina Ruggiero

**Affiliations:** ^1^Clinical Nutrition Unit, IRCCS Ospedale Policlinico San Martino di Genova, Genova, Italy; ^2^Department of Informatics, Bioengineering, Robotics and Systems Engineering, University of Genova, Genova, Italy; ^3^Phoniatric Unit, IRCCS Ospedale Policlinico San Martino di Genova, Genova, Italy

**Keywords:** cohesiveness, viscosity, viscoelasticity, dysphagia, aspiration pneumonia, texture-modifies food, thickeners

## Abstract

Dysphagia is a swallowing disorder characterized by the difficulty in transferring solid foods and/or liquids from the oral cavity to the stomach, imparing autonomous, and safe oral feeding. The main problems deriving from dysphagia are tracheo-bronchial aspiration, aspiration pneumonia, malnutrition and dehydration. In order to overcome dysphagia-induced problems, over the years water and food thickening has been used, focusing specifically on viscosity increase, but limited results have been obtained. Elastic components and their effects on the cohesiveness on the bolus should be taken into account in the first place. We provide an analysis of dysphagia and suggest possible corrections to the protocols which are being used at present, taking into account rheological properties of food and the effect of saliva on the bolus. We reckon that considering such aspects in the dysphagia management market and healthcare catering would result in significant clinical risk reduction.

## Introduction

Oropharyngeal dysphagia (OD) is a dysfunction of the digestive system, consisting of a difficulty in swallowing which affects the proper transit of the bolus in the upper digestive tract. The main complications of dysphagia are tracheo-bronchial aspiration (food transit in the airways with choking), aspiration pneumonia (pneumonia caused by food in the lungs), malnutrition and dehydration. Dysphagia can be a result of neurological disorders (such as stroke, Parkinson's disease, multiple sclerosis) or muscular disorders (such as metabolic myopathy, muscular dystrophy, myasthenia gravis). It can relate to solid foods only, to semi-liquid or liquid foods, and also to various different consistencies. Dysphagic patients fail to control food flow, which is fast and turbulent (1, 2), through the oropharynx, with the risk of misdirection through the respiratory tract.

A strategy that has been usually implemented over the years to overcome this problem is the use of thickened foods, according to the belief that the transit time of modified consistency food products is higher than normal and this gives the glottis more time to close (3). At present, dietary treatment for dysphagia has focused to a great extent on fluid thickening by viscosity increase (4). It could be expected that chronic dysphagia patients would improve using thickened fluids (with nectar, honey, and pudding consistency) and specially made and nutritional enriched texture modified foods (pureed and minced). Even thought, it seems to be difficult to increase dietary intake with pureed and thickened food (with unspecified consistency) for elderly people with chronic dysphagia when small, frequent meals are provided (5). It is felt that patients with OD are likely to obtain a better dietary intake with texture-modified foods rather than with normal food. However, Foley et al. (6) found that there is a lack of evidence on the importance of texture modified foods and thickened fluids related to the amount of diet intake for adults with acute dysphagia. Moreover, Andersen et al. found that the evidence supporting the use of texture modified foods and thickened fluids is not strong and that more studies are needed for the management of dysphagia (7). It is also important to take into account that there is no evidence for texture modified foods and thickened fluids about the prevention of aspiration pneumonia in patients with chronic dysphagia (8–10). In some clinical conditions, the risk of aspiration can also be reduced by chin-down or chin-tack procedures, in which the chin and the neck are approached, making tongue base and epiglottis closer to the posterior pharyngeal wall (11). In any case, the approach to the problem of dysphagia treatment must be supported by counselling and individual education regarding the consistency of the food, normal, and abnormal swallowing and safe swallowing techniques for the prevention of aspiration.

The National Dysphagia Diet (NDD) published in 2002 by the American Dietetic Association (12) provides guidelines about thickened dietary supplements. Its recommendations have been challenged in many respects. After one year from its publication, Mc Callum pointed out that the four categories of NDD guideline are vague and impractical in clinical applications (13). According to Quinchia et al. there would not be scientific evidence or rationale given by NDD for the temperature and shear rate chosen for the scales. Furthermore, these scales only considered viscous properties and elasticity was not even mentioned (14). Some researchers think that the NDD definitions are “obscure definitions” and believe that the guideline can't be used easily in real applications (15). Optimum rheological properties of food and drink for a specific kind of dysphagia are only unsatisfactorily known and have not been effectively validated (16). However, the NDD is described as the most creditable guideline in the literature. The most important lack of this guideline is considering only one rheological parameter (i.e., single point viscosity) as classification criterion. Nowadays, modern rheological instruments and techniques should allow to review this guideline (17).

More recentely, there are other concerns about the use of thickened foods. In the food industry, hydrocolloids are widely used to modify the sensory properties of foods to obtain a specific viscosity and palatability. While all hydrocolloids are able to thicken and give viscosity to aqueous solutions, not all hydrocolloids are able to structure themselves in such a way as to form a three-dimensional network that can contain the solvent (gel). In the case of thickening, the biopolymers used cause non-specific entanglement which, above a certain concentration, results in an increase in stickiness. On the other hand, gels are composed of polymeric molecules that form an interconnected cross-linked network that provides the system with increased rigidity (18). The thickening of nutritional mixtures using hydrocolloids mostly derives from long polymer chains that get entangled together when their concentration increases. In diluted systems, these molecules can move freely and viscosity decreases. In this respect, after the intake of a thickened food mixture, saliva dilutes it and breaks it up, making viscosity decrease considerably. This inconvenience occurs specially when using starch-based thickeners, because of α-amylase, an enzyme contained in saliva that breaks the chains of amylose and amylopectin that are components of the starch. The use of gums for food thickening can mitigate but not entirely eliminate unwanted viscosity reduction. Oral processing of food (especially if prolonged as in the case of patients suffering from dysphagia) still leads to a reduction in viscosity even when gum-based thickeners are used. These aspects should be considered in the management of dysphagia therapy, to prevent patients receiving food with inappropriate consistency, taking into account the physician's instructions (19–21).

From a clinical point of view, viscosity modification has reduced the risk of aspiration, while the preparation of minced and pureed foods is irrelevant for this risk. Special attention should be paid to the change in the cohesiveness of these foods after homogenization and other methods that result in a plurality of physical changes. The present work focuses on the cohesiveness of the bolus in this respect.

## Rheological properties of food

Sometimes, from a clinical point of view, the administration of a thickened mixture can achieve limited benefits, especially when an appropriate posture is not associated (e.g., in case of delayed pharyngeal response, reduced posterior motility of the base of tongue etc). Foods normally exhibit a viscoelastic behaviour and, in order to improve food cohesiveness, viscoelasticity has been recently recognised as crucial to improve swallowing and decrease the risk of “breathing food” in the airways. Cohesiveness is a mechanical feature that is part of textural properties of the food. It can be defined as the strength of the internal bond making up the body of the sample (22). Therefore, scientific efforts should focus on the production of higher-cohesive mixtures using substances that produce an increase in cohesiveness forces (H-bonds, van der Waals, electrostatic, hydrophobic, and hydrophilic forces), among the non-aqueous components of the solution rather than focus on increasing viscosity due to the exchange of moments between molecules. In this respect, it is also important to note that cohesiveness, like other physical properties, can be based on more basic rheological foundations and, specifically, is related to the elastic modulus (23). In order to improve dietary therapy for dysphagia not only the viscous component (loss modulus) has to be considered, but also the elastic component (storage modulus) has to be taken into account. In this respect, rheological properties, as mechanical properties that affect deformation and material flow in the presence of stress, play a key role. The Japanese dysphagia modified diet classification paid attention to the hardness, adhesiveness, and cohesiveness of the foods (24). Specifically, easy to swallow foods are defined as a texture that satisfies three criteria: (1) under 15,000 N/m2 in hardness, (2) under 1000 J/m2 in adhesiveness, and (3) between 0.2 and 0.9 in cohesiveness (25, 26).

The aim of rheology is the measurement of the properties of materials that affect their behaviour (deformation and flow) when they are subjected to external forces. In recent years much work concerning food rheology has been carried out (27, 28) and can provide study and application opportunities in many respects.

Food is primarily made out of biopolymers and watery arrangements containing broken up sugars and macromolecules, such as for example, proteins, polysaccharides, and lipids from an extensive variety of plant and animal sources. Moreover, water is a main part of foods and plays a noteworthy part in the formation of edible structures and in their stockpiling steadiness.

The characterization of thickened food is very important in order to identify key parameters to improve the formulation of dysphagic foods by evaluating the contribution that different hydrocolloids can make to edible mixtures. In general, the rheological measurement techniques that are used can be divided into three groups: fundamental, imitative, and empirical. An overview on these techniques is shown in Table 1.

**Table 1:** Rheological measurement techniques.

**Categories**	**Definition**	**Instrumentation/test**	**Advantages**	**Disadvantages**
*Fundamental*	Measure rheological properties like viscosity and elasticity	Dynamic oscillator rheometer Creep test Stress relaxation test	Parameters are physically well defined Measurements are reproducible Useful for structure-property relationship	Expensive equipment Poor correlation with sensory Slow to perform
*Imitation*	Mimic the condition to which the food is subjected during eating/processing	Texture Profile Analysis Farinograph Visco-Amylo-Graph	Closely duplicate mastication or sensory methods Good correlation with real situation	Measures parameters which are often poorly defined, but appear to relate to textural quality
*Empirical*	Stimulates the conditions to which materials are subjected in practice	Puncture and penetration test Extrusion test Flow funnel Bostwick or Adam consistometers	Good correlation with real situation Easy and fast to perform Inexpensive equipment	Measured parameter are poorly defined Arbitrary procedure

Fundamental techniques can measure well-defined rheological properties. This category includes dynamic oscillator rheometry (storage and loss modulus measurement), creep test, and stress relaxation test. Dynamic oscillator rheometry is used for storage and loss modulus measurement and is the most common dynamic method for viscoelasticity measurements; creep test can measure a strain as a function of time when a constant stress is applied; stress relaxation test observed the decrease in stress in response to the same amount of strain applied. Fundamental measurements provide exactly what it is measured, but are slow and the instrumentation is expensive.

Imitation methods are less common and imitate the conditions to which food is subject in practice. The instrumentation that can be used are the texturometers [Texture Profile Analysis (TPA)] which provide multiple parameters (such as modulus, hardness, brittleness, adhesiveness, elasticity/springiness, and cohesiveness). These methods give a complete measurement of texture, but are not suitable for routine work.

Empirical tests measure properties that are not well-defined, but may be related to experience with properties of interest (e.g., Bostwick or Adam consistometers). Unfortunately, these tests are dependent on both equipment and sample geometry, so it may be difficult to compare data between different samples. To this category belong the tests proposed by IDDSI (29), which are intended to provide a standard terminology by using some rapid and easy to perform tests. The major advantages of empirical tests are the simplicity and rapidity of performing, on the other hand the disadvantages are that the procedures are arbitrary and it is not possible to convert data to other systems.

### Viscosity and viscoelasticity

**Figure 1:**
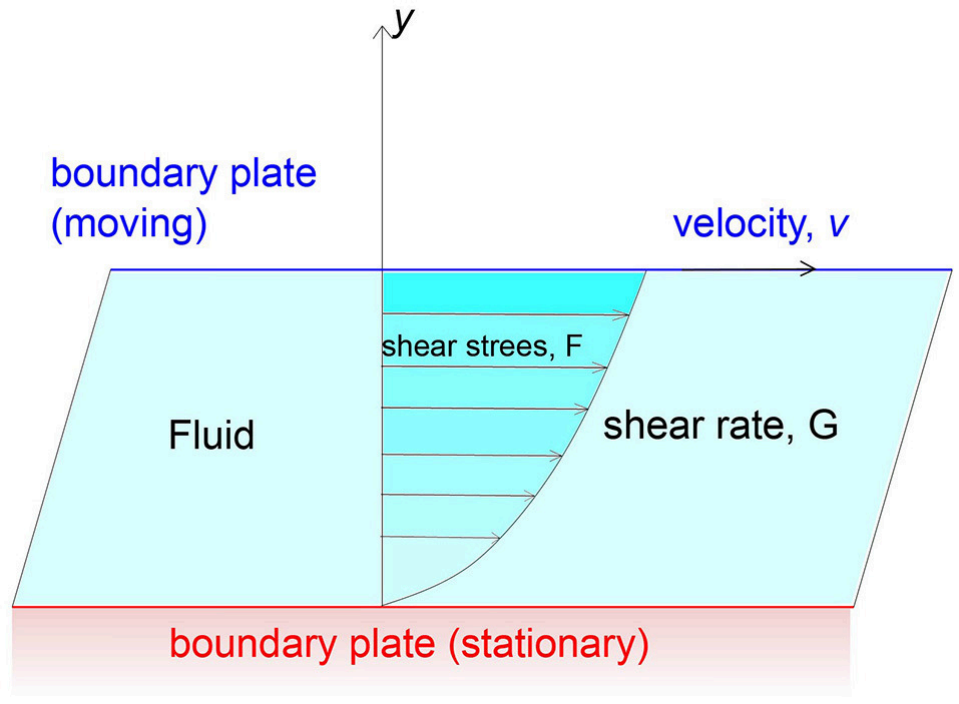
Representation of Viscosity as a constant relating an applied shear stress to the resulting shear velocity.

Viscosity can be defined as the resistance of a fluid to flow and is due to the intramolecular cohesion forces that attract molecules together and to the exchange of moments between molecules. More specifically, we can consider a fluid between two large parallel plates, one of which is boundary and the other is moving with a velocity *v* in the *x* direction. The fluid can be regarded as made of infinitesimal layers (Figure 1). The layer closer to the moving plate will move in the *x* direction at a velocity almost equal to *v*, conversely the layer next to the stationary plate will move very slowly. Considering a fluid with a very large number of layers, the sliding among layers generates a velocity gradient that it is called shear. The relationship between the frictional force f applied to a surface of area A and velocity gradient d*v*/d*x* was stated by Newton and is summarized by

$$\begin{array}{l} f=\eta A\left(\frac{dv}{dy}\right) \end{array}$$

where η is defined as coefficient of viscosity or simply viscosity; *f* /*A* = *F* is the shear stress and d*v*/d*y* = *G* is the shear rate. When η is a constant, the fluid is called Newtonian otherwise the fluid is non-Newtonian and η is a function of *F* or *G*.

Several guidelines have been adopted for dysphagia treatment, but normally only food viscosity is taken into account. (12, 30). In these guidelines, foods are clustered into groups based on viscosity that is commonly measured at shear rate of 50 s^−1^ as a representative shear rate for the mouth cavity, however there is a lack of convention about the shear rate used to define viscosity (31). It is also important to note that on the basis of sensory viscosity analysis it has been estimated that the shear rare during swallowing varies greatly and are within a range of 5 to 1000 s^−1^ (28). This high variation makes guidelines based on cutting forces of 50 s^−1^ unrealistic. Viscosity is constant and independent from the shear rate for Newtonian fluids only, and considering only a single shear rate in the swallowing process is too simplistic (32). For a non-Newtonian fluid viscosity is also called apparent viscosity because it is possible to define it for each value of shear rate. If we consider a non-Newtonian fluid (as most of the foods) viscosity changes when the shear rate changes. Specifically, viscosity decreases for shear thinning fluids (e.g., concentrated fruit juice, melted chocolate, salad dressing) and increases for shear thickening fluids (e.g., corn starch suspensions or some particular types of honey (Eucalyptus ficifolia, Eucalyptus eugeniodes) (33). Shear thickening fluids are found much more rarely. This behaviour of non-Newtonian fluids is known as viscoelastic and in this case, fluids show both elastic and viscous components.

**Figure 2:**
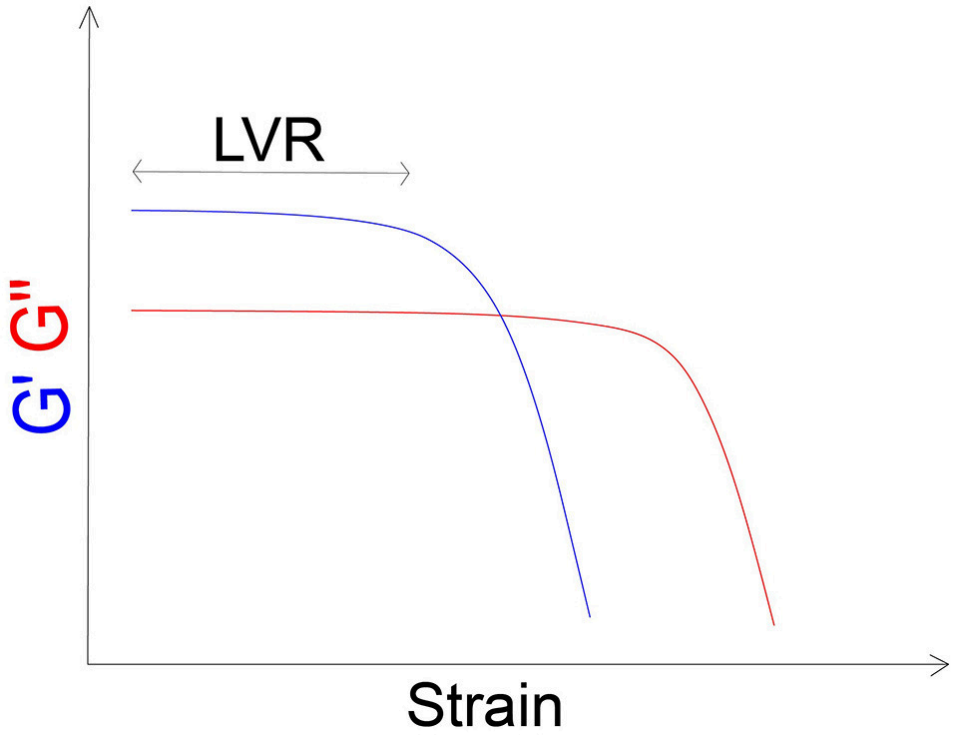
Example of representation of storage (G′) and loss (G″) moduli vs. the applied strain. The LVR is clearly identifiable.

A viscoelastic material shows both elastic properties and viscous properties at the same time. Viscoelasticity can be characterized as linear or non-linear viscoelasticity. For linear viscoelasticity, the rheological properties depend only on time and not on the magnitude or the stress that is applied, while for non-linear viscoelasticity the mechanical properties of the material are influenced by the time and by the magnitude of the stress. The range in which materials show a linear behaviour is defined as linear viscoelastic region (LVR). Normally, food materials show linear viscoelastic activities if they are tested in low stress conditions (linear range), and non-linear behaviour for large deformations (Figure 2). The linear range can be determined by experimental measurements. Chewing and swallowing processes may involve high deformations outside the LVR, therefore the investigation of food samples in that region is also important. The study of non-linear viscoelasticity is much more complex than linear viscoelasticity and complex calculations are needed to characterize sample behaviour in the non-linear region (34), mostly when the composition of the bolus is complicated. In the LVR the relationship between stress and strain can depend only on the rate of deformation that introduces a time dependence characterizing the viscoelastic behaviour. If the strain stress ratio depends not only on time, but also on the strain applied (usually for high strain values) it is said that the behaviour is viscoelastic non-linear.

The complex modulus G^*^ is a property of viscoelastic materials and it is composed of two parts: the storage modulus *G*′ that represents the elastic component of the material and the loss modulus *G*″ which characterizes the viscous behaviour

$$\begin{array}{l} G^{*}=G^{\prime }+iG^{\prime \prime } \end{array}$$

where *i* is the imaginary unit.

Small Amplitude Oscillatory Shear Testing (SAOS) can be used for the measurement of linear elastic and viscous components of materials and can be performed using a rheometer, an instrument capable to measure the stress linked with a specific deformation or motion. The test is performed applying a sinusoidal strain and measuring the corresponding stress on the sample. The stress response σ to the sinusoidal strain γ is expressed by

$$\begin{array}{l} \sigma \left(t\right)=\gamma_{0}G^{\prime }\left(\omega \right)\sin\left(\omega t\right)+\gamma_{0}G^{\prime \prime }\left(\omega \right)\cos\left(\omega t\right) \end{array}$$

Another property that is often used is the loss tangent, which expresses the relative aspects of the viscous and elastic components and is given by

$$\begin{array}{l} \tan\delta \left(\omega \right)=\frac{G^{\prime \prime }\left(\omega \right)}{G^{\prime }\left(\omega \right)} \end{array}$$

**Figure 3:**
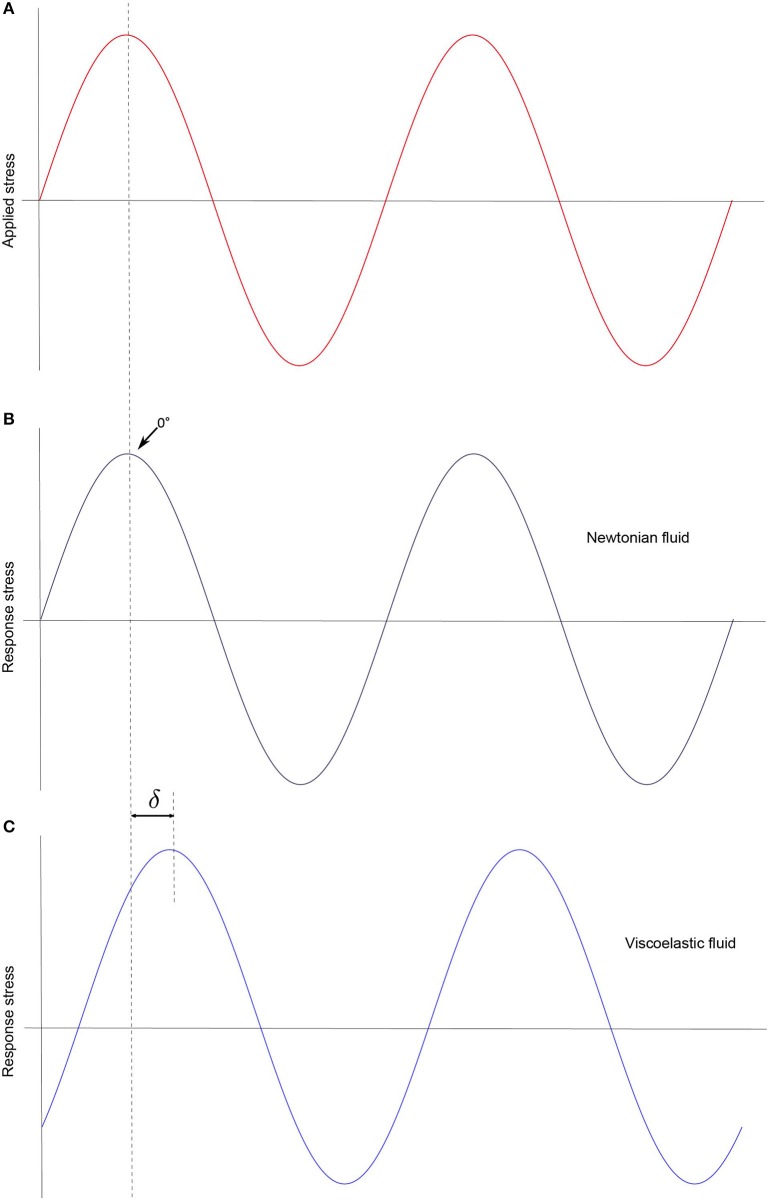
In SAOS testing a sinusoidal strain is applied **(A)**. As result, a response stress can be measured. δ can identify the sample properties. If the response stress is in phase with the applied strain (δ = 0) the fluid is Newtonian (no elastic component) **(B)**, otherwise the sample is viscoelastic **(C)**.

δ represents the phase difference between the applied strain and the response stress. In the case of a fully elastic sample δ is equal to 90°, while in the case of Newtonian fluids the applied stress and the response are in phase (δ = 0; Figure 3).

The texture modification of minced or pureed foods by agents that modify viscosity only is often used, but is not highly effective. The effects of viscoelastic properties and of other rheological parameters, such as yield stress (the stress in which the material starts to deform plastically) and structure recovery, on swallowing along with sensory evaluation of dysphagia-oriented products and the effect of saliva on the bolus should be assessed for non-Newtonian fluids, such as minced, and pureed foods (17).

In food analysis, measurements are very often carried out only in the LVR, in which viscoelastic moduli are well defined and with specific physical information. Measurements in the LVR very often do not correlate with sensory and oral processing data (35). It is possible to measure material behaviour beyond LVR using the large amplitude oscillatory shear (LAOS) technique (36), which exploits the protocol developed by Ewoldt et al. (37) and then validated by Melito et al. (38), in which the higher order harmonics are analysed by the Fourier transform. Although the LAOS technique has been widely used in the study of polymers and their properties, its use in the food industry does not seem to occur today, and very few studies have been carried out as relates to the non-linear viscoelasticity of foods (39) and of products for dysphagics (14).

### Extensional flow

A property that should be considered is the elongation behaviour of food mixtures. During the pharyngeal phase of swallowing the bolus is subjected to extensional deformations. Extensional viscosity can be defined according to the type of deformation the material undergoes and can be uniaxial, biaxial, or planar. In the simplest case of uniaxial deformation, when the material is extended in one direction at the rate of $\overset{{}^{\circ}}{\varepsilon }$, it undergoes a symmetrical contraction in the other two dimensions equal to half of the set deformation ($\overset{{}^{\circ}}{\varepsilon }$/2). Extensional viscosity may be expressed as

$$\begin{array}{l} \eta_{e}\left(\overset{{}^{\circ}}{\varepsilon }\right)=\frac{\sigma_{xx}-\sigma_{zz}}{\overset{{}^{\circ}}{\varepsilon }} \end{array}$$

Where E is the velocity (V) gradient

$$\begin{array}{l} \overset{{}^{\circ}}{\varepsilon }=\frac{\partial V}{\partial x}\;\left(in\;uniaxial\;deformation\right) \end{array}$$

For Newtonian fluids a coefficient, known as “coefficient of viscous traction,” has been determined that binds shear viscosity with extensional viscosity by Trouton (40). For these fluids, it can be noted that the extensional viscosity turns out to be three times the shear viscosity. For viscoelastic materials, however, extensional viscosity follows the Trouton rule only for small extension values, while for high deformation values the Trouton ratio can be very high. Bearing in mind the fact that for a Newtonian fluid *G*′ = 0 occurs, it is evident that the elastic component plays a fundamental role with respect to the extensional behaviour of a non Newtonian fluid, even if it is not possible to consider separately the viscous and elastic effects due to the non-linearity of the materials (41). Although extensional viscosity has not been much discussed so far for dysphagia, an additive that can provide a high extensional viscosity may contribute to bolus cohesiveness during the swallowing process (42).

### The role of saliva

Saliva is a watery substance secreted by salivary glands and contains a mixture of proteins, electrolytes, and small organic compounds. Saliva secreted in absence of stimuli is called unstimulated saliva, whereas saliva produced by a stimulus is stimulated saliva. It is worthy to be noted that the composition of saliva and its physical properties may vary depending of the type of the stimulus. Saliva has high elasticity and low viscosity. Moreover, different types of stimulation can lead to different features of saliva. Mechanically stimulated saliva is more watery and less elastic than the one produced by an acid solution stimulus. (43). Extensional properties of saliva seem to be performed by mucin glycoprotein (44). Saliva has a pivotal role for the formation of a “swallow-safe” bolus, besides lubrication that is its primary rheological function (45). The mouth has a large number of receptors, including texture receptors (46), and acts as a rheometer: when foods are eaten, mastication, and mixing with saliva occur until the bolus is regarded as safe to be swallowed (47).

### Bolus properties for a safe swallow

Researchers during the years have qualitatively identified the bolus properties for a safe swallow. Peyron et al. (48) observed an increase in adhesiveness, springiness and cohesiveness, and on the contrary a decrease in the bolus particle size distribution during the masticatory process. Cohesiveness can be defined as the force that binds particles of a substance together and is the molecular attraction exerted across a surface within liquid or solid that resists internal rupture. The role of saliva in increasing cohesiveness is fundamental. Human mucin components MUC5 and MUC7B (glycosylated proteins) naturally secreted in food stimulated saliva (49, 50) determines bolus formation by ensuring food aggregation and cohesion and increases viscoelastic properties (51).

Chen et Lolivret argue the importance of bolus extensional stretch-ability for the swallowing (52), Prinz et al. by a mathematical model focused on particle size reduction and viscosity of saliva, pointed out the importance of a peak of cohesive forces between the particles (53). Gray-Stuart defined as key property of the bolus its volume and deformability. Precise measurement of bolus swallowability is difficult and not yet developed, even though a first attempt has been made by Ng et al. (54).

Specifically, measurements of viscoelasticity and cohesiveness of the bolus and also of texture-modified food (non-Newtonian fluids obtained by transformation of foods with saliva) would be advisable.

## Discussion and conclusions

Despite the fact that all guidelines for the preparation of dysphagic meals are only referred to food viscosity before it is introduced into the mouth, it is also important to take into account that the mixing of saliva with foodstuff may change its rheological properties. Although few analyses regarding viscosity and thickening after the mixing with saliva have been performed (19, 21, 55), these findings must be taken into account in dietary prescriptions for dysphagia.

Leonard et al. show that the risk of aspiration in dysphagic patients significantly decreases when gum-based thickeners are used, and even though the elastic moduli were not considered, an increase in G′ due to the presence of the gum can be hypothesized (56). Specifically, even though very little information is available about thickened food characterization related to viscoelastic properties, it has been found that guar gum may contribute to an increase of storage modulus (57). Similar results can be achieved using xanthan gum thickener (58). The fundamental difficulty in measuring the rheological properties of food is that every individual—healthy or suffering of dysphagia—has different physiological characteristics (such as masticatory function or salivary secretion) that can affect the measurement and lack reproducibility (59). However, it is also interesting to note that an excessive increase in elasticity leads to an increase in the difficulty of swallowing the bolus (60). This must be taken into account for the identification of elasticity and viscosity values that can lead to an optimal formulation of the food. It is also important to point out that for equal concentration of thickening agents, the different food condition, such as temperature or higher salt content, may lead to a significant variation in the rheological properties of the resulting thickened food (61). It can be inferred that the bolus cohesivity plays an essential role in safe bolus safety and swallowing. The exclusive attention on the viscosity aspect in foodstuff preparation for dysphagia treatment is reductive and may not be sufficient to prevent pulmonary aspiration.

In a highly cohesive material it is more difficult to separate the particles than in a low cohesive material. According to Cichero, cohesiveness of the bolus conditions the initiation of the swallow rather than just the size of the particles of food/fluid. In the oral phase, chewing and mixing with saliva as a binding agent is useful to produce a bolus that is both lubricated and cohesive (62). Nowadays, there is not a robust and standardized methodology, acknowledged by the scientific community as a valid way of evaluating cohesiveness (63, 64).

From a literature analysis, it can be noted that although dietary changes in the consistency of the foods and fluid thickening improve the nutritional state of patients with chronic dysphagia, the reduction of the risk of aspiration pneumonia remains debatable (7, 65). Deceptively, expert stakeholders (industries and clinical rheologists) have underlined the need to increase viscosity as the main target of dietetic intervention in dysphagia and suggest that many minced or pureed food could be mixed with xanthan gum or modified maltodextrines amylase resistant. In this respect, the evaluation of cohesiveness of semiliquid and semisolid is mandatory.

It is worth noting that in stroke patients it would appear that consistency modification mediated by the use of traditional thickeners is of equal risk with respect to the individual choices of the dysphagic ones. Finally, individual counselling on swallowing strategies seem to be of great importance. Individual counselling with a joined follow up, about texture-modified food and thickened fluid, may decrease the risk of aspiration pneumonia in people with acute dysphagia (66). Moreover, the education of people with chronic dysphagia to the use of “chin-down” technique can reduce the risk of aspiration pneumonia as much as eating foods with modified consistency (67). However, individual counselling together with the prescription of texture-modified foods and thickened fluids, seems to be no better than self-chosen consistency of food. Specifically, there is no evidence as relates to a reduction of the risk of aspiration pneumonia in people with chronic dysphagia (68). The lack of effectiveness of modified consistency food might be attributed to a systematic error due to the use of substances that are inappropriate or not suitable for improving cohesiveness and viscoelasticity in LVR and also in non LVR. These features are crucial for improving swelling of the bolus. The structural changes in food mixtures should be oriented to a flake off minimization of the bolus. Moreover, because of compression and elongation of the bolus, it will be easier to travel through a site where mobility and speed of transit are impaired for neuromuscular reasons (reduction of penetration and suction in the respiratory tract).

Even from a diagnostic point of view, evaluations based on viscosimetric data (since nutritional mixtures behave as non-Newtonian fluids) cannot contribute to the investigation of cohesiveness, and therefore the information generated by viscosimetry is only partial (7). Moreover, recent findings show that reaching a certain degree of viscosity at 50 s^−1^, as suggested by many guidelines for meal preparations for dysphagic patients, is not satisfactory in order to avoiding the risk of aspiration and allowing a safe swallowing (69) and can affect breathing-swallowing coordination (70). Unfortunately, to date, a robust technique for measuring cohesiveness is still lacking. Even if the TPA test using double compression is often used, there is a reduced correlation between measurement and sensory perception (64). It could be useful to carry out new studies of viscoelasticity and cohesiveness of products for dysphagia and thickened food especially in non LVR using LAOS technique.

Many studies have been carried out with regard to xanthan gum and guar gum and their use as thickening agents for the preparation of dysphagic foods. Only few studies have involved other types of thickeners (hydrocolloids) which are commonly used as additives in the food industry (18, 71). Moreover, the use of a single type of polysaccharide thickeners may not be optimal to meet all requirements for dysphagic foods. One solution would be to prepare mixtures whose synergistic effect could improve viscous and elastic bolus characteristics and maintain them sufficiently also within the LVR, both for obtaining safe food for swallowing and for improving palatability and flavour release from the food (72). In this respect, another disadvantage of treating dysphagia with thickened foods is that many patients are not satisfied with meals with modified consistency and their consumption on a daily basis (73). Furthermore, while many tests have been carried out on different foods, even at different temperatures, there is very little evidence of rheological properties of the bolus immediately before swallowing, completely neglecting the activity of saliva in the oral cavity. Moreover, it would be important to evaluate thickeners not only on healthy volunteers, but also on dysphagic patients who may have different salivary compositions that could differently affect the rheological behaviour of the bolus.

**Figure 4:**
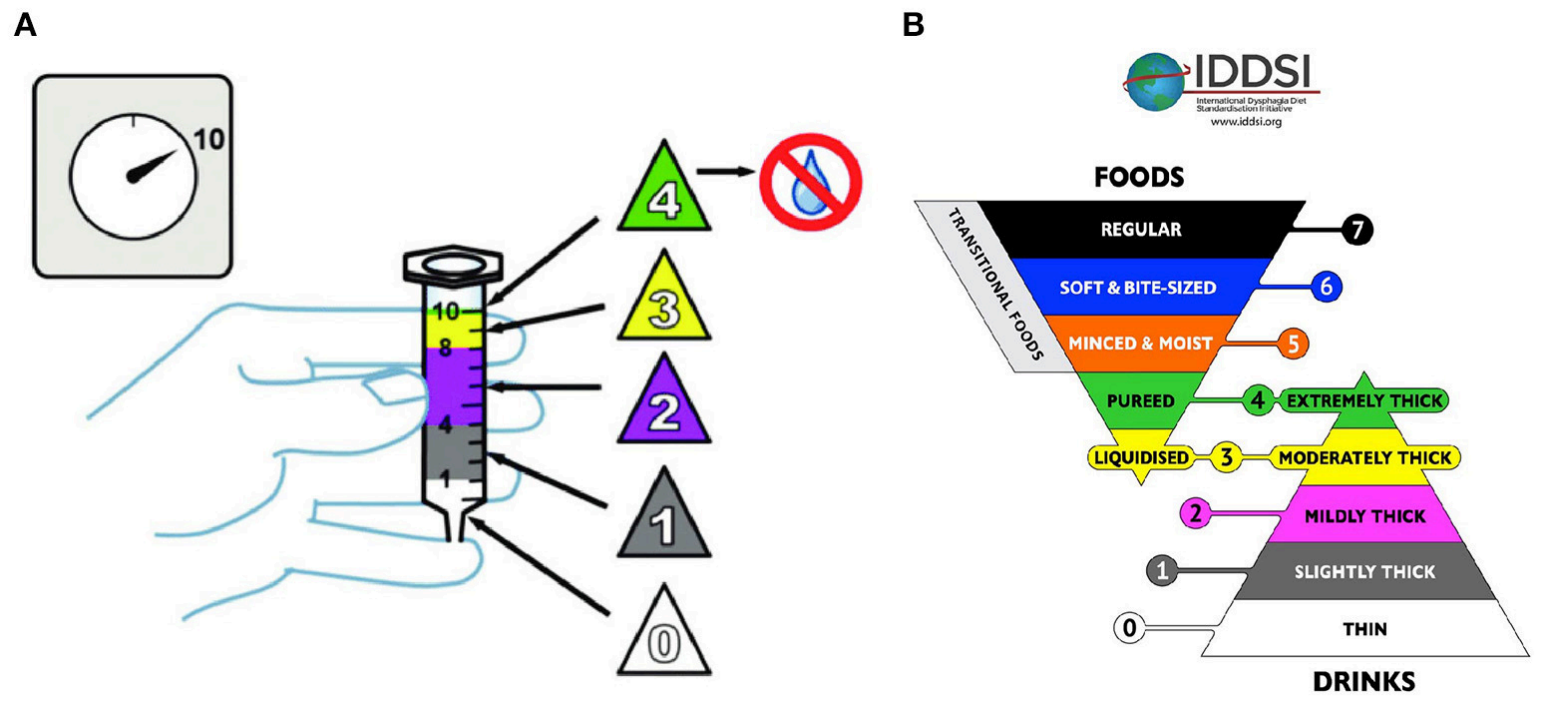
The IDDSI flow test, consisting of filling a syringe of 10 ml of fluid and verifying how much fluid leaves the syringe kept upright in 10 s **(A)**. Five levels are defined: level 0 the syringe completely drains; Level 1 remain between 1 and 4 ml; Level 2 between 4 and 8 ml; Level 3, between 8 and 10 ml; Level 4, the syringe remains full. At these levels is associated the corresponding terminology **(B)** (29) (CreativeCommons Attribution Sharealike 4.0 Licence).

A further problem is the lack of standardization. The IDDSI framework (29) has introduced a standardization of the terms associated with the consistency of food on the basis of simple empirical tests that can be implemented in the hospital or at the patient's home (Figure 4). However, a classification based on rheological properties of the food (that can be adapted to each patient phenotype based on the degree of the disease) has not yet been considered. It would also be useful to develop clinical trials for the quantitative definition of optimal food cohesiveness, which at present derives exclusively from careful observation of the tolerance of different types of foods.

The problem of evaluating products for dysphagia from the bedside must be reversed to the production of the products by industry and catering companies. Therefore, empirical analyses (i.e., IDDSI), which are valid where practical conditions need to be easy and simple to assess, should be replaced by more rigorous analyses, which should be carried out upstream, i.e., in the industry or in large industrial kitchens typical of the conservative food industry. In the food industry, rheological characteristics have to be defined to guarantee the quality perceived by the consumer and to ensure the mass production of repeatable and standardised products. In this respect, additional quality criteria and clinical guidelines based on rheological methodologies will need to be introduced for industry and health-care catering in order to ensure the reduction of clinical risk associated with dysphagic diets, giving indications to the clinician who must decide on these patients.

## Author contributions

SS and NM contributed equally to conception and design of the study. NM wrote the first draft of the manuscript. SS and NM wrote sections of the manuscript. All authors contributed to manuscript revision, read, and approved the submitted version.

### Conflict of interest statement

The authors declare that the research was conducted in the absence of any commercial or financial relationships that could be construed as a potential conflict of interest.
